# Effect of Probiotics and Herbal Products on Intestinal Histomorphological and Immunological Development in Piglets

**DOI:** 10.1155/2020/3461768

**Published:** 2020-04-24

**Authors:** Daiga Gāliņa, Līga Ansonska, Anda Valdovska

**Affiliations:** ^1^Latvia University of Life Sciences and Technologies, Lielā iela 2, Jelgava, LV-3001, Latvia; ^2^Institute of Food Safety, Animal Health and Environment “BIOR”, Lejupes iela 3, Rīga, LV-1076, Latvia

## Abstract

The aim of the study was to evaluate the effect of probiotics and herbal products on the intestinal histomorphological and immunological development in piglets. Accordingly, 2-week-old piglets were allocated in 4 groups: C (basal diet), Pro (basal diet + probiotics), Pro+B (basal diet + probiotics + buckwheat bran), and H (powder of herbs). After 6 weeks of the experiment, 4 piglets from each experimental group were randomly selected and slaughtered at a slaughterhouse. Samples of tissue and digestive content from the jejunum and colon were collected for bacteriological, histological, and immunohistochemical examination. The results showed that probiotics increased the number of *Lactobacillus* spp. in the small (*p* < 0.05) and large intestines. The intestinal histomorphology was improved (*p* < 0.05) in all experimental groups by an increased villus height, VH : CD ration, colon crypt depth, and number of Ki-67^+^ epithelial cells. A higher number (*p* < 0.05) of goblet cells and their acidification were observed in group Pro, while the density of goblet cells was decreased by the herbs. Probiotics increased (*p* < 0.05) the number of intraepithelial lymphocytes (IELs), density of CD3^+^ cells in Peyer's patches (PPs), and lamina propria (LP). In group H, a dual effect on the CD3^+^ cell distribution was observed. The herbs reduced (*p* < 0.05) the number of IELs and CD3^+^ in LP but increased the distribution of CD3^+^ cells in PPs. In the colon, herbs increased CD3^+^ cells in LP as well. It suggests that probiotics and herbs had influence on the intestinal histomorphology and the ability to modulate the mucosal immune system; however, the combination of probiotics and buckwheat bran was not so convincing, probably due to the inhibitory effect of the buckwheat bran on the probiotics used.

## 1. Introduction

Weaning is a critical period in the life of pigs; factors such as separation from the sow, a new environment, and dietary changes promote a negative effect on the growth of piglets. Moreover, the dominance of the intensive farming model, the increase of the size of piglet groups, and limited space result in injuries and spread of diseases among pigs. It is a great challenge for the mucosa of the gastrointestinal tract and immune system that are not fully mature in this life period of pigs [1]. In addition, reduced feed intake affects negatively the tropism and morphology of the intestinal mucosa in the weaning period [2]. These circumstances may increase the necessity for antimicrobials; however, they promote the spread of resistant bacteria [3]. Despite the fact that the use of antibiotics as feed supplements has been banned for food-producing animals, antibiotic resistance is a high priority for the policy of the EU, and high levels of resistance for several bacterial species are still being observed [4]. Antimicrobial resistance is a serious global threat; therefore the development of alternative feed supplements is important to prevent the selection and transmission of resistant bacteria.

The gastrointestinal tract of pigs is a very important immunological competent organ. Therefore, immunological development of piglets may be an effective intervention aiming at not only the reduction of the use of antibiotics but also improvement of the production performance and a higher return on input for swine producers [5]. The maturation of gastrointestinal tract and development of immunity depend on the composition of the indigenous microbiota [6]. The intestinal microbiota has an essential role in introduction, training, and functioning of the host immune system, but, on the other hand, the immune system has to keep the symbiotic relationship of the host with those various microbes [7]. Moreover, microbiota can affect intestinal morphology, that way improving the intestinal development, health, and functionality [8]. It has been reported that the gut microbiota can be influenced by dietary means using different feed supplements such as prebiotics, probiotics, and herbal products [9].

Probiotics have been extensively studied, and a number of positive effects on piglets have been observed: the increased dominance of healthy microbiota, reduced shedding of pathogens and disease symptoms, promoted digestive capacity, improved maturation of the intestinal tissues, and improved immune responses [9, 10]. Although the effects of the probiotics have not been generalised, there are several factors on which they depend, e.g., the variation in the used microbial strains, doses applied, duration of treatment, and husbandry practices [5]. Buckwheat is an important functional food. Its proteins are particularly rich in lysine, arginine, and aspartic acid; besides, it is also rich in many rare components, e.g., flavones, flavonoids, phytosterols, and d-fagomine [11]. D-Fagomine has a eubiotic effect on the intestinal microbiota; it promotes diversity in the gut microbiota [12]. Natural bioactive compounds produced from plants have a positive effect on the growth and health of animals [13]. Herbs and their products have antimicrobial, anti-inflammatory, and antioxidative properties; they promote digestibility and immunity; however, their application in the diet has still been mostly based on their antimicrobial effects [14]. The medicinal benefits of plantain have been well known around the world for hundreds of years. Plantains contain flavonoids, alkaloids, terpenoids, iridoids (aucubin, catalpol), fatty acids, phenolic acids, and vitamins; several studies have suggested that the plantain has an important role in the management of ulcers, bacterial and viral infections, pain, inflammation, and diarrhoea [15]. The nettle is rich in bioactive compounds and nutrients; its extract inhibits proinflammatory cytokine production, decreases the level of C-reactive protein, and increases superoxide dismutase, thereby demonstrating its high anti-inflammatory and antioxidant effects [16]. The main bioactive components of St. John's wort are hypericin and hyperforin. These bioactive compounds are well known as antidepressants, and, in addition, their antibacterial, antiviral, and anti-inflammatory properties have been observed as well [17].

Probiotics and herbals have many positive properties; they can be very prospective, but their usage in pig farming has been increasing slowly, mainly due to their variable results, possible local irritation of some plants or their products, and unclear way of actions to take. Therefore, the purpose of the present research study was to evaluate the effect of probiotics and herbal products on the intestinal histomorphological and immunological development in piglets.

## 2. Materials and Methods

### 2.1. Preparation of Feed Additives

Probiotics, herbal powder, and buckwheat bran were used as feed supplements. Probiotics containing various strains of lactic acid bacteria and yeasts were commercially available as “ProbioHelp” (Baltic Probiotics, Latvia). Buckwheat (*Fagopyrum esculentum* L.) bran was selected from an organic farm in Latvia. The herbal powder was made by the authors of this article using nettle leaves (*Urtica dioica* L.), plantain (*Plantago major* L.), and flowering tops of Saint John's wort (*Hypericum perforatum* L.). During July 2017, plants were collected at Dobele and Livani districts of Latvia, and each plant specimen was authenticated by the Institute of Horticulture of the Latvia University of Life Sciences and Technologies. The plants were dried at a room temperature in a shady, well-ventilated space and after that grounded to powder by a mechanical grinder.

### 2.2. Experimental Design and Sample Collection

The experimental design involved 44 piglets (Duroc × Landrace) at two weeks of age. The piglets were allocated in four groups (11 piglets into a pen), named as groups C, Pro, Pro+B, and H, respectively. Control (group C) received a basal diet, group Pro received a basal diet supplemented with probiotics, and group Pro+B received a basal diet supplemented with probiotics and buckwheat bran, but group H received a basal diet supplemented with herbal powder. The combination of probiotics was an addition to the drinking water depending on the age of piglets: the 2, 3, 5, 6, and 7 weeks old received the concentrations of 1%, 0.75%, 0.45%, 0.34%, and 0.32%, respectively; however, the buckwheat bran and herbal powder were included in the basal diet in constant concentrations of 3% and 1.5%, respectively. All of the piglets were given a basal diet and water through a local feeder and nipple drinker. At the end of the experiment, 4 piglets from each experimental group were randomly selected and slaughtered at a slaughterhouse. Their tissue samples (about 1.5-2 cm) of the jejunum (20 cm proximal from the ileocecal fold (*plica ileocaecalis*)) and colon (proximal part of *colon descendens*) were collected. The samples were rinsed with cold physiological saline (0.9% w/v) and fixed in 10% neutral buffered formalin. The contents of the same sites of the jejunum and colon were collected in sterile containers and put in a cooler box. The content samples were transported to the laboratory within 2 hours for bacteriological examination.

### 2.3. Enumeration of Bacteria

The contents of the jejunum and colon were used for determination of the count of *Enterobacteriaceae*, *Escherichia coli*, and *Lactobacillus* spp. The initial and serial dilutions were made in peptone saline diluent (Maximum Recovery Diluent, Biolife) according to ISO 6887-1:1999. For the isolation and enumeration of *Enterobacteriaceae* and *Escherichia coli*, Violet Red Bile Glucose agar (Biolife) and Tryptone Bile X-Gluc agar (Biolife) were used according to ISO 21528-2:2007 and ISO 16649-2:2007, respectively. For isolation and enumeration of *Lactobacillus* spp., MRS agar with Tween 80 (Biolife) was used according to the manufacturer's instructions for this medium. The colony-forming units were calculated and expressed as log_10_ colony-forming units per gram of digestive contents.

### 2.4. Slide Preparation for Histology and Immunohistochemistry

Formalin fixed tissues were trimmed and passed in cassettes. Then the tissues were dehydrated in increased concentrations of alcohol solutions, embedded in paraffin, and sectioned using rotary microtome. From each sample, several slides were prepared, and each of them contained two 3 *μ*m sections.

### 2.5. Intestinal Histomorphology Analysis

The tissue sections were stained with haematoxylin and eosin [18]. After that, they were analysed by the light microscope (Leica DM3000LED, Germany), and the images were taken by a camera (Leica DFC450, Germany). The obtained images were processed using an image processing and analysis system (Leica Application Suite, Version 4.10.0). For morphological measurements, the villus height, villus width, crypt depth, and the villus height to crypt depth ratio were analysed using 100x magnification. A total of ten well-oriented villus-crypt structures were selected and measured in triplicate for each sample. The villus height was measured from the tip of the villus to the mouth of the crypt. The crypt depth was measured as the lowest point of the invagination between the adjacent villus to the mouth of the crypt and the villus width at the middle of the villus. The total count of goblet cells was calculated in the structures of villus and crypt, as well as the goblet cell units of the villus height and crypt depth. Histopathological features (grade of inflammation, inflammatory cells, parasites, and bacteria) for each experimental animal were analysed using 100x–1000x magnification.

### 2.6. Neutral and Acidic Mucin Staining

Periodic-Acid-Schiff (PAS) and alcian blue (AB, pH 2.5) staining techniques [18, 19] were used for the detection of the neutral and acidic mucin secreting goblet cells, respectively. The total number of mucin secreting goblet cells was counted by evaluating 10 villus and crypts for each group. The density of mucin secreting goblet cells was calculated as the number of goblet cells per micrometre of the villus height and crypt depth. For the analysis, 400x magnification was used.

### 2.7. Immunohistochemistry (IHC)

In IHC staining, specific antibodies of CD3 (Dako, A0452) and Ki-67 (Dako, clone MIB-1, IR621) were used for the identification of T-lymphocyte and cellular marker for proliferation, respectively. Briefly, sections were placed on IHC microscope slides (APTACA, 1804532) and fixated for 1 hour at 60°C. Then the sections were deparaffinised, rehydrated, and rinsed with deionised water. Epitopes were retrieved by HIER buffer (Target Retrieval Solution (10x), pH 9, Dako, S2367) in a microwave (350 W–15 min and 750 W–7 min) to the boiling temperature for three times. The endogenous peroxidase activity was blocked by incubation for 10 minutes with peroxidase blocker (Dual Endogenous Enzyme Block, Dako, SM801) and followed by rinse with TBS buffer (Wash Buffer 20x, Dako). Primary antibodies of CD3 and Ki-67 were added and incubated for 60 min. Antibody of CD3 was added at a dilution of 1 : 200, but Ki-67 was ready to use. After incubating, the primary antibodies were rinsed by TSB buffer (Wash Buffer 20x, Dako, K8000), and their binding was detected with HRP system (EnVision™ + Dual Link System-HRP (DAB+), Dako, K8010). The sections were incubated for 45 min and rinsed by TBS buffer (Wash Buffer 10x, Dako, K8000). Diaminobenzidine (DAB+, Dako, K8010) was added for visualization by the light microscopy as brown-coloured reaction of positive structures. Finally, the sections were counterstained with haematoxylin and mounted with a coverslip. For analysing IHC, 100–400x magnification was used. The numbers of proliferating epithelial cells (Ki-67^+^) and intraepithelial T cells (CD3^+^) were counted in the crypt and villus, respectively, and expressed as the number of positive cells per 100 *μ*m. The relative frequency of CD3^+^ cells was analysed in the lamina propria (separate per zones of the crypt and villus) of the jejunum and colon, in Peyer's patches (separate per zones of intrafollicular and dome + folicullar) and in submucosa of the colon. Ten randomly selected visual fields were analysed for each sample.

### 2.8. Statistical Analysis

R Studio software (version 0.99.489) was used for the statistical analysis. The effect of treatment on bacterial populations and histomorphology of the jejunum and colon in piglets was analysed with one-way ANOVA; Duncan's multiple range test was used for post hoc identification of significant differences among the groups treated. The relative frequency of CD3^+^ cells was determined according to the semiquantitative counting method [20]. The relative frequency of positive structures in the view field was marked as follows: (0)—no, (0/+)—occasional, (+)—few, (+/++)—few to a moderate amount, (++)—a moderate amount, (++/+++)—a moderate to numerous amount, and (+++)—abundant; the obtained data was transformed to rank 0, 1, 2, 3, 4, 5, and 6 for statistical analysis, respectively. Kruskal-Wallis H test and Conover-Iman post hoc test were used for nonparametric analysis of variance. The results were expressed as the mean ± SEM. The statistical significance was considered at *p* < 0.05. Values of *p*=0.05 − 0.10 were reported as tendencies. Microsoft Excel 2016 version (16.0.4266.1001) was used for the visual representation of the data obtained.

## 3. Results

### 3.1. Intestinal Microbiota

The diet supplemented by probiotics increased the number of *Lactobacillus* spp. in the jejunum (*p* < 0.05) and colon, compared to groups C and H. The herbs had a tendency (*p*=0.064) to decrease the number of *Enterobacteriaceae* in the colon. The number of *Escherichia coli* was lower in group H, but the difference was not significant (Table 1).

### 3.2. Intestinal Histopathology

**Figure 1:**
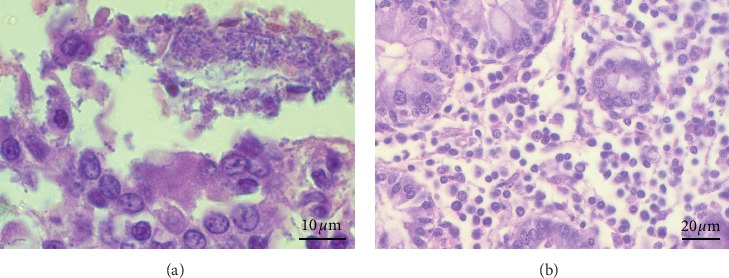
Histological features in the jejunum and colon of piglets: hematoxylin and eosin technique. (a) The colon section has shown a minimal quantity of bacteria on the surface of mucosa, 1000x magnification. (b) The colon section has shown a mild infiltration of inflammatory cells in the lamina propria, mainly consisting of lymphocytes and plasmocytes, 400x magnification.

In the piglets of all experimental groups, a minimal to mild chronic diffuse enterocolitis was found with a minimal to mild infiltration of inflammatory cells, mainly consisting of lymphocytes and plasmocytes (Figure 1(b)). In addition, in jejunum, a moderate infiltration of eosinophil leukocytes was found, but in separate crypts of the colon a minimal infiltration of neutrophil leukocytes was observed. Eosinophil leukocytes were more located in the lamina propria (LP) within the part of the crypts and were observed slightly more in the supplemented groups. In all groups, some villi were blunted and fused with minimal to moderate desquamation of epithelium. Furthermore, mild desquamation was observed in superficial part of mucosa in the colon in all experimental groups. In jejunum, a minimal amount of bacteria was detected on the surface of villi in all groups, but the presence of bacteria in the crypts was observed only in groups Pro and Pro+B. Similarly, in the colon, minimal to moderate quantum of bacteria was observed on the surface of mucus (Figure 1(a)), while in the crypts, again, it was observed only in groups Pro and Pro+B. Of all experimental groups, in few crypts of the colon, there was a minimal invasion of cryptosporidium, slightly more in groups H and C, but no significant differences were found between the experimental groups.

### 3.3. Histomorphological Measurements of Intestine

The basal diet supplemented with herbs, probiotics, and their combination with buckwheat bran increased the villus height in groups H (+35%, *p* < 0.05), Pro (+17%, *p* < 0.05), and Pro+B (+12%, *p* < 0.05) compared to group C. The villus width was decreased in group C (*p* < 0.05) compared to all of the supplemented groups. In jejunum, supplementation of feed additives had no significant effect on crypt depth. The villus height to the crypt depth (VH : CD) ratio was increased (*p* < 0.05) in groups H, Pro, and Pro+B compared to the control group. In the colon, the crypt depth was observed to be higher (*p* < 0.05) in groups Pro, Pro+B, and H compared to group C (Table 2).

### 3.4. Goblet Cells and Their Secreting Mucins

In jejunum villi, the lower density of goblet cells was observed in group H (*p* < 0.05), while the total number of goblet cells was not affected. In contrast, in the colonial crypts, the total number of goblet cells was higher in groups that received probiotics but did not affect the density of goblet cells (Table 3).

**Figure 2:**
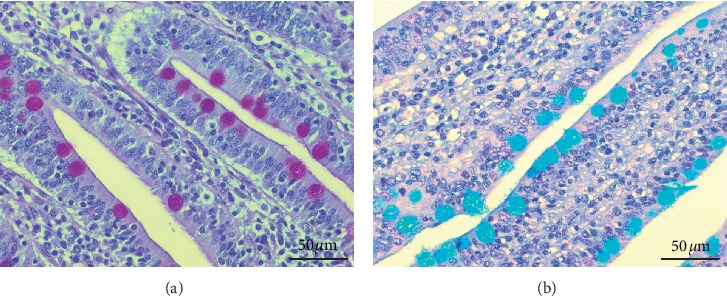
Goblet cells in the jejunum of piglets, 400x magnification. (a) Periodic-Acid-Schiff technique, positive cells in red. (b) Alcian Blue technique, positive cells in blue (pH 2.5).

In jejunum, inclusion of probiotics in the basal diet increased (*p* < 0.05) the total number of AB^+^ goblet cells in the villi and crypts, but the herbals decreased (*p* < 0.05) the density of AB^+^ goblet cells in the villi. In the colon, the total number of PAS^+^ goblet cells in group C (*p* < 0.05) decreased, but the total number of AB^+^ goblet cells was higher in group Pro (*p*=0.03) (Table 4 and Figures 2(a) and 2(b)).

### 3.5. Immunohistochemical Analysis of Cell Proliferation and Infiltration of Lymphocytes

**Figure 3:**
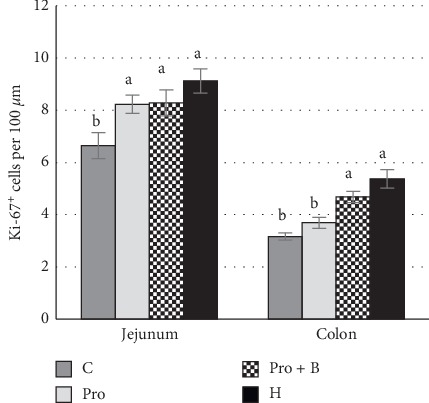
Ki-67 positive epithelial cells in crypts of the jejunum and colon. (a, b) Mean values with different letters are significantly different (*P* <0.05). The error bars show the standard error of measurements (ten randomly selected visual fields of 4 piglets from each group (*n* = 16)).

The Ki-67^+^ cells dominated in jejunum crypts and in the germinal centres of Peyer's patches (PPs), but in colon Ki-67^+^ cells dominated in the deeper parts of crypts. In both jejunum and colon, few to moderate expressions of Ki-67 positive cells were seen additionally in the inflammatory cells present in LP. In jejunum, all of the supplemented groups (*p* < 0.05) had a higher number of Ki-67+ enterocytes compared to the control group, but in the colon, a significant effect of enterocytes proliferation was observed in groups Pro+B and H (Figure 3).

**Figure 4:**
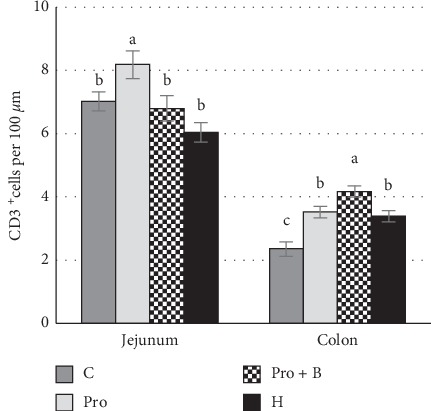
The number of intraepithelial (IE) CD3^+^ T cells in the jejunum and colon. ^a,b^Mean values with different letters are significantly different (*p* < 0.05). The error bars show the standard error of measurements (ten randomly selected visual fields of 4 piglets from each group (*n* = 16)).

**Figure 5:**
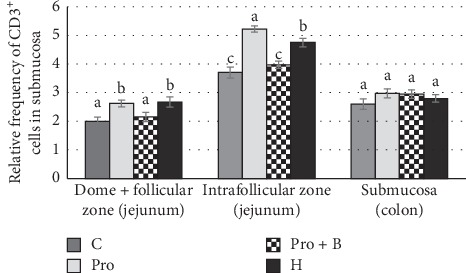
Relative frequency of CD3^+^ cells in Peyer`s patches of the jejunum and in the submucosa of colon by administration of herbals, probiotics, and probiotics combination with buckwheat bran. ^a,b^Mean values with different letters are significantly different (*p* < 0.05). The error bars show the standard error of measurements (ten randomly selected visual fields of 4 piglets from each group (*n* = 16)).

**Figure 6:**
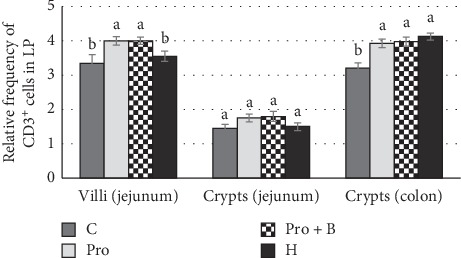
Relative frequency of CD3^+^ cells in the lamina propria (LP) of the jejunum and in the colon by administration of herbals, probiotics, and probiotics combination with buckwheat bran. ^a,b^Mean values with different letters are significantly different (*p* < 0.05). The error bars show the standard error of measurements (ten randomly selected visual fields of 4 piglets from each group (*n* = 16)).

**Figure 7:**
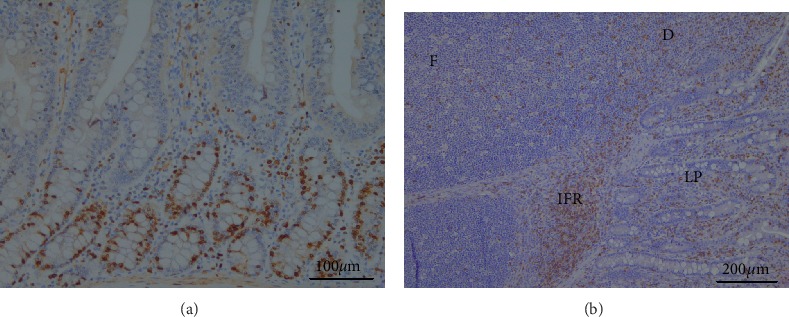
IHC. (a) Proliferating epithelial cells (Ki-67^+^ cells) in crypts of jejunum, 200x magnification. (b) CD3^+^ T cells in different parts of jejunum. F: follicular zone; D: dome; IFR: intrafollicular zone; LP: lamina propria; 100x magnification.

The CD3^+^ cells findings in the intestine were variable. Considering the differences between the supplemented groups, in jejunum, the total number of intraepithelial CD3^+^ cells was higher (p<0.05) in group Pro, but in colon - in groups Pro B, Pro andH, compared to group C (Figure 4). Numerous amounts of CD3^+^ cells were observed in the intrafollicular zone of PPs (Figure 5). Moderate to numerous amounts of CD3^+^ cells were observed in the LP of jejunum villi and colon crypts (Figure 6). Amount of CD3^+^ cells in dome region was classified as few to moderate, but it was rare in the germinal centres of PPs (Figure 7(b)).

In PPs, probiotics and herbs increased the relative frequency of CD3^+^ cells (*p* < 0.05) in both intrafollicular zone and dome + follicular zone. No differences were observed in the distribution of CD3^+^ cells in submucosa of the colon (Figure 5). Groups Pro and Pro+B increased (*p* < 0.05) relative frequency of CD3^+^ cells in the part of villi in LP. Groups Pro, Pro+B, and H increased (*p* < 0.05) distribution of CD3^+^ cells in LP of the colon (Figure 6). The IHC positive cells (Ki-67^+^ and CD3^+^) are showed in Figures 7(a) and 7(b).

## 4. Discussion

The intestinal epithelial and immune cells with the mucus layer provide the first barrier against indigenous microbiota, pathogens, and external antigens. Indigenous microbiota attached to mucus prevents colonisation pathogens by occupying empty niches, stimulates the mucosal defence mechanisms, and maintains homeostasis of immune response [21]. Weaning is a very critical period for piglets; it causes imbalance of the microbiota and intestinal mucosal barrier dysfunction, resulting in the intestinal inflammation and diarrhoea [22].

The main condition for the piglet health is a healthy intestinal microbiota. Intestinal microbiota has several very important roles, for example, taking part of digestion, fermentation of carbohydrates, promoting maturation of the intestinal mucosa, protection from pathogens, production of vitamins and taking part in induction, and training and functioning of the immune response [23]. Our results have demonstrated that probiotics increased the number of *Lactobacillus* spp. in small and large intestines. The ability of probiotics to increase the count of *Lactobacillus* spp. in piglets has been well documented [5]. The combination of probiotics and buckwheat bran slightly decreased the count of *Enterobacteriaceae* and increased the count of *Lactobacillus* spp. but did not reach even an equivalent level of *Lactobacillus* spp. as it was observed when using only probiotics. Some authors have confirmed that buckwheat possesses the activity of prebiotics [24, 25], which can improve the intestinal microbiota by increasing the count of lactic acid bacteria and decrease the count of *Enterobacteriaceae* [24] and *E. coli* [9, 26]. On the one hand, the count of *Enterobacteriaceae* may decrease due to acetates, lactates, and bacteriocins produced by lactic acid bacteria [27]. On the other hand, in the buckwheat bran, the seed coat contains a high content and diversity of phenolics, such as epicatechin, procyanidin B2, epicatechin gallate, rutin, catechin, isovitexin, hyperoside, and isoquercetin [28] with antimicrobial activity [29, 30]. Therefore, probably, the antimicrobial activity of buckwheat bran was not only aimed at reducing *Enterobacteriaceae* but also could influence the activity of the supplemented probiotics, preventing the increase in the number of *Lactobacillu*s spp. In our study, the selected plant combination was focused on the reduction of the count of *Enterobacteriaceae* without significantly affecting the number of *Lactobacillus* spp. Many herbs have antibacterial activity, which encourages their use as alternatives to antibiotics for piglets diet [14]. Although the structure of Gram-positive bacteria is responsible for the fact that they are more sensitive to antibacterial activity of most herbs, indigenous microbiota uses pili to bind extracellular matrix proteins to form a biofilm, which makes them more resistant in the host organism [14, 31]. Therefore, the combination of nettle, plantain, and St. John wort could succeed in the modulation of gut microbiota, with the aim of reducing the number of pathobionts, without affecting the indigenous microbiota.

In the histopathology examination, a minimal to mild chronic diffuse enterocolitis was observed in all piglets of the experimental groups. Changes in dietary and environmental factors by weaning cause a significant morphological and functional alteration in the gastrointestinal tract, known as weaning-associated intestinal inflammation. In the postweaning period, piglets have characteristic chronic enteritis with a variety of inflammatory cells, especially mononuclear cell infiltration in LP [32, 33]. The authors of this research study observed eosinophil leukocytes infiltration in LP, although the presence of parasites in the small intestine was not detected. It has been reported before that infiltration of eosinophils is a characteristic observed in healthy pigs without parasite infection [34]. Research studies have detected that eosinophils are not only a significant part of inflammatory effector cells of parasitic or allergy infection but can also function as immunomodulatory cells [35]. In turn, infiltration of neutrophil leukocyte in some crypts of colon was associated with a mild invasion of cryptosporidium [36]. Histopathology results reveal bacterial spread on the surface of mucus and in the crypts. Some authors insist that intestinal microbiota forms biofilms above mucus outer layer in normal conditions [37]. Low feed intake after weaning and lack of enteral nutrition cause acute changes in gut physiology and impact indigenous microbiota [38]. Accordingly, fragments of biofilm and pathobionts cross the mucus barrier and adhere to the epithelial cells and translocate through the cell paracellularly. Pathobionts activate host immunity and cause inflammation [37]. However, there may be another explanation for the presence of bacteria in the crypts. Some authors point out that several bacteria, including *Lactobacillus* spp., are capable of surviving close to the epithelial surface and even colonize the villi and crypts. The presence of bacteria in these specific niches may be crucial to repopulate initial bacterial community in gut lumen following environmental challenges [31]. In piglets that received probiotics, minimal to moderate location of bacteria in the crypts was observed more often. Since the main component of probiotics is different species of *Lactobacillus*, it could be the reason for the ability of probiotics to promote the attachment of beneficial microbiota to these specific niches. As no significant histopathological findings were observed between the experimental groups, minimal to mild chronic diffuse enterocolitis in all groups could be a logical step of bowel maturation, caused by new diet and circumstances.

Parameters of small intestinal histomorphology, such as the villus height, crypts depth, and their ratio, are important informative indicators on gut health status [39]. A higher villus ensures a greater count of enterocytes, which increases the surface area and promotes several positive effects: higher enzyme production, increased absorptive area, and improved system of nutrient transport [40]. Crypts are locations of epithelial stem cells that are responsible for proliferation of epithelial cells. Proliferation and differentiation of enterocytes provide coverage and growth of villi; in addition, they play a key role in the local and systemic immune responses [41, 42]. VH : CD ratio is often used for objective measures of histological changes. A higher VH : CD ratio is regarded mainly positively, because an absorptive surface increases and the tissue turnover rate decreases [43]. Our study demonstrated that inclusion of herbs in the basal diet increased the villus height and VH : CD ratio. Similar to our results, other researchers have reported about beneficial effects of herbs on gut histomorphology in piglets [8] and poultry [44]. The effects of plants or essential oils on intestinal histomorphology are mainly due to the decreased bacterial load in the gut [45]. Besides the positive effect of herbs on the intestinal villi height and VH : CD ratio, we also observed greater width of the villi. Windisch et al. [46] assumed that herbal essential oils can have a dual phytogenic action: on the one hand, they are able to decrease pathogen pressure and thereby improve the intestinal surface area; on the other hand, most of essential oils can cause irritation of intestinal tissues, and, as a result, the intestinal surface area can be reduced. The probiotic supplement to the basal diet improved the villus height and increased the VH : CD ratio, thereby positively affecting the development morphology of the small intestine. The beneficial effect of probiotics can result in their colonisation of niches in the intestines, thereby reducing the count of pathogens. The reduced toxicity and increased nutrient absorption can contribute to the growth and restoration of the villi [47]. In jejunum, the crypt depth was not affected by the feed supplements; nevertheless, a higher density of KFi-67^+^ enterocytes was observed in all supplemented groups. A similar effect was observed after oligosaccharide feeding [48]. In turn, deeper crypts were observed on histomorphology of the colon in all supplemented groups. Based on the fact that buckwheat and polysaccharides of herbs exhibit prebiotic properties [23, 49], lactic acid bacteria utilise them into SCFAs, particularly acetate, propionate, and butyrate, which are an important energy source for growth of colonocytes [50]. Moreover, butyrate is an active inhibitor of inflammation and stimulates the regeneration of mucosa [51].

The small and large intestines contain specialized cells, called the goblet cells. They secrete large glycoproteins that form the mucus layer and cover the epithelial cells. There is a single mucus layer in the small intestine and there is a two-layered system in the large intestine; in addition, the inner is dense and practically sterile, while the outer is loose and makes an ecosystem of commensal bacteria [52]. It is the first frontline of innate host defence against exogenous and endogenous irritants and microbial adhesion and invasion, but, at the same time, it allows the exchange of water, gases, and nutrients [53]. Mucins are divided into neutral and acidic subtypes. Oligosaccharide chains of neutral mucin contain glucose, galactose, mannose, or fructose, while acidic mucin usually involves both groups of sialic acid and sulphate acid, and the final categorisation determines the groups that are predominant [54]. Both acidic and neutral mucins increase the viscosity of the mucus layer for epithelium protection, while acidic mucins protect against bacterial translocation [55]. We observed that probiotics increased the amount of goblet cells in the large intestine and influence their differentiation by increasing acid mucin production in the small and large intestines. The ability of probiotics to influence the proliferation and differentiation of goblet cells has been previously reported. In germ-free mice, the count of goblet cells in the small intestine increased after their conventionalisation by faecal microbiota [56]. Challenged with enterotoxigenic *E coli* (ETEC) the count of goblet cells in the piglet intestines decreased but increased in piglets that had orally received a moderate dose of *Bacillus probiotics* mixture as pretreatment, before being challenged with ETEC [57]. In growing-finishing pigs, probiotics increased the number of goblet cells in all examined intestinal crypts; furthermore, an increase in acidic producing goblet cells in the duodenum and colon was observed [58]. Several scientists have tried to explain how microbiota and probiotics can alter proliferation of goblet cells and promote acidification of mucin. Bacteria colonize the mucus and use the mucin molecules as carbon, nitrogen, and energy sources [59].They release end-products of mucus fermentation, different secretory metabolites, and bioactive factors, which activate diverse signalling cascades and secretory elements and effect goblet cells. For example, during recent years, scientists have drawn attention to the role of microbiota to release proteolytic enzyme, meprin *β*, which is anchored in the apical membrane of enterocyte. Meprin *β* diffuses into the mucus and after that it cleaves and releases MUC2 from the goblet cells attachment [60]. Moreover, bacterial structural elements, such as lipopolysaccharides (LPS), flagellin A, and lioteichoic acids (LTA), or several metabolites (SCFA, adenosine triphosphate) are able to regulate mucin gene expression by affecting the host immune responses. [61]. For example, *γδ* T cells have an important role to modulate the intestinal mucus layer by impacting the goblet cell function and mucin expression. Therefore, the decreased count of *γδ* T cells is responsible for decreasing the count of goblet cells and reducing mucin containing sialic acid [62]. Our results showed that probiotics increased the number of intraepithelial lymphocytes (IELs) in the small intestine. In pigs, IELs belong to *δγ* T cell population [63]. Probiotics structural elements (LPS, LTA) are important stimulators to innate and adaptive immunity [61] and may be responsible for an increased number of IELs. Therefore, the higher number of goblet cells and acidification of mucus can be explained by the immunostimulatory effect of the probiotics used; however, at the same time, the direct effect of probiotics on goblet cells should not be excluded. The effect to increase goblet cell count and modulation of mucin by using a combination of buckwheat bran and probiotics was not so convincing compared to the usage of probiotic alone. This may be due to the inhibitory effect of buckwheat bran on the probiotics used. The herbs reduced the density of goblet cells; furthermore, both acidic and neutral mucin producing goblet cells were decreased in the small intestine. Currently, there are no reports on the direct impact of plants on the number of goblet cells; nevertheless, a lower microbiota load decreases the number of goblet cells and their size [64]. The antimicrobial activity of herbs has been studied widely; although the data are various, most of them recognize the inhibiting effect against coliforms, *Escherichia coli* and *Chlostridium perfringens* [45]. Based on our previous research study, we state that this herbal mixture reduces the count of *Enterobacteriaceae* and *E. coli* in the postweaning period of piglets [26]. Presumably, the effect of our herbal mixture on the density of goblet cells may be explained by its antimicrobial activity. Furthermore, a slightly reduced level of IELs in the small intestine indicates an inhibitory effect on immune cells caused by herbs. The suppression of mucosal immunity may also be a reason for the reduced numbers of goblet cells and mucin production.

Based on IHC results of our study, we can state that probiotics increased the proliferation of epithelial cells (Ki-67^+^) in the crypts of small intestine, and the herbs and combination of probiotics and buckwheat bran increased proliferation in both the small and large intestines. Some authors have observed that probiotics can destabilize the interaction of enterocytes with the basal membrane and increase proliferating enterocytes in crypts, resulting in the increase of the rate of repopulation of epithelial cells in the small intestine. In addition, there is a probability that some strains of probiotics may increase the cell proliferative effect by interacting with intestinal stem cells [65]. Recent research papers have highlighted the critical role of lactate in the development of intestinal stem cells. Proliferation of intestinal stem cells was stimulated after lactate binding to receptor Gpr81 and causing Wnt/*β*-catenin signals of Paneth and intestinal stromal cells [66]. Comparatively, administration of probiotics alone, or their combination with buckwheat bran, showed that this combination significantly increased epithelial cell proliferation not only in jejunum but also in colon. Buckwheat bran is a significant source of fibre [67]. Dietary fibre can alter the microbial population and impact production of SCFA in a pig model. However, the main producer of SCFAs is indigenous obligate anaerobes microbiota. Most probiotics indirectly increase SCFAs, too. The proximal part of the large intestine, lactic acid bacteria, produces lactate, which is a substrate for the production of acetate, propionate, and butyrate by several SCFA-producing indigenous bacteria [68, 69]. SCFAs absorbed in the colon are as fuel for the mucosal epithelial cells [70]. It suggests that additional inclusion of fibre in the pig diet increased the production of lactate and SCFAs, thus increasing proliferation of colonocytes. Interestingly, the highest number of epithelial cells proliferation was observed after administration of herbs. Several research reports point out that herbal extracts are able to influence cells proliferation and differentiation. The polysaccharides and hyperforin of *Hipericum perforatum* induce and stimulate differentiation of keratinocytes *in vivo* and *in vitro* [71, 72]. Astragalus polysaccharides stimulate proliferation, migration, and differentiation of intestinal epithelial cells [11]. Considering the important role of SCFAs to improve physiological and morphological parameters of intestines, several reports have been written about the effects of herbal medicines on SCFAs production in the gut. Herbs contain many glycosides and carbohydrates that can be a substrate for production of SCFAs [73]. Polysaccharides of the *Plantago asiatica* L seeds significantly increase the concentration of the total SCFA and propionic and butyric acid in rats [74]. Based on the previous reports and our studies, our findings suggest that the proliferation of the intestinal cell epithelium is mainly affected by the end-products of microbial metabolism, but it does not exclude the direct effect of certain bioactive components on cell proliferation, particularly when herbal medicines are administered. Therefore, characterising the chemical compounds of herbal medicines, especially polysaccharides, is a critical step to understand the mechanisms of herbal medicines.

Another important part of the intestinal barrier is the mucosal immune system that protects host's gastrointestinal tract against pathogens by using gut-associated lymphoid tissue (GALT). It forms one of the largest parts of the immunological tissues in the body [75] and includes organised tissues (mesenteric lymph nodes, Peyer's patches (PPs), and scattered immune cells in LP) and epithelial cells known as IELs [76]. Special attention has been paid to distribution of T cells. T cells play a critical role to provide intestinal homeostasis and activation of immune response to pathogens [77]. We observed that probiotics increased the distribution of T cells in PPs, LP, and IELs. In the studies of germ-free animals, it is proven that microbiota plays the main role in developing the host's immune system. In germ-free animals, the PPs have been reduced, having a significant shortage of T cells in them. Moreover, a reduced number of immune cells are observed in the tissues [78]. There are several immune modulators that are associated with microbiota and the most important of them are toll-like receptors and SCFAs [79]. The ability of probiotics to increase frequencies of T cells in PPs has been reported previously, but the balance between effector and regulatory T cells was not changed [80]. In our study, probiotics increased T cell distribution not only in intrafollicular zone (T cell zone) of PPs but also in the dome and B cell follicular zone. Unique subsets of CD4 T cells are located there: follicular regulatory T cell (Tfr) and follicular helper T cell (Tfh). Tfh has an important role in the formation of germinal centres (GCs); it promotes differentiation of B cells to plasma and memory B cells [81], while Tfr suppresses humoral immunity [82]. More detailed studies of Tfh and Tfr distribution ratio are needed to estimate the ability of probiotics to influence the humoral immunity. However, previous studies have pointed out that probiotics increase the number of IgA positive cells in PPs and, thereby, increase protection against mucosal pathogens. IgA suppresses bacterial binding to epithelial cells and acts against toxins [49]. After being inducted in the PP, mature T cells migrate to LP and between epithelial cells. The LP consists mainly of CD4^+^ T cells [63]. CD4^+^ T cells represent a different collection of subsets with specific cytokines and chemokines that can activate other immune cells, resulting in activated or suppressed immune responses [77]. Nevertheless, after probiotics administration, the increased number of T cells in LP is often associated with an increased Treg [48, 80]. Several reports about the ability of probiotics to increase the count of IEL in the small and large intestines of piglets have been published [33, 83]. In pigs, IELs are mainly CD8^+^ [72, 84]. Under homeostatic conditions, IELs regulate the noninterruption turnover of epithelial cells. They destroy the infected cells and stimulate cell proliferation by producing a keratinocyte growth factor [85]. IELs play an important role in a mucosal injury or attack of pathogens. They produce cytokines and chemokines that rapidly destroy pathogen-infected or damaged target cells [77]. In mice with reduced IEL, an increased number of bacteria in mesenteric lymph nodes were observed [86]. Therefore, the inclusion of probiotics in piglet feed not only modulates cell mediated immunity and impacts humoral immunity but also improves the intestinal barrier by increasing cell epithelium proliferation and regulation of mucus layer. The main effect of the combination of probiotics and buckwheat bran was observed in the large intestine by increased T cell population in LP and IELs. Given that buckwheat bran is a fibre source, primarily, it is a source of energy for the intestinal microbiota. Without being digested in the small intestine, it reaches the large intestine, where microbiota decomposes nondigestible fibres to SCFAs. As it is known, SCFA can not only modulate beneficial bacteria but also activate the intestinal immune system. Although the effect to increase the number of *Lactobacilli* by the combination of buckwheat bran and probiotics was not observed, the effect on other beneficial bacteria such as *Bifidobacteria* cannot be excluded. Furthermore, prebiotics can show direct effect on the immune system without any modulation of intestinal microbiota [87]. Interestingly, the probiotics included in this combination did not increase IEL in the small intestine as it was observed when used alone, which could indicate the inhibitory effect of buckwheat bran on the included probiotics. In our study, herbs increased T cell population in both intrafollicular zone and dome and B follicular zone, which points to immune-modulating activity against the cells in Peyer's patches. Several studies have found that polysaccharide of different plants can stimulate immune-competent cells in PPs [88]. Interestingly, after administering the herbs, the number of T cells in LP and IEL depended on the location side of the intestine: in jejunum, a tendency to slightly decrease the number of T cells was observed, while a significant increase of T cell density in the colon was present. Most plants contain molecules that are able to moderate the activity of immune cells; moreover, the same plant may have both stimulatory and inhibitory effects depending on the circumstances. The water extract of plants contains more hydrophilic components, such as polysaccharides, which can tend to activate the immune system responses. In contrast, ethanol or methanol extracts of plants contain hydrophobic compounds such as flavonoids and terpenoids, which usually inhibit the immune cell responses [89]. In our study, herbs decreased IELs in jejunum. Several authors have reported about the impact of herbs to decrease IELs [90]. On the one hand, most of herbs have antibacterial properties, which allow them to be used as antibiotic alternatives [14]. Herbs modulate or reduce microbial load; they decrease activation of toll-like receptors followed by decreased proliferation of T cells [79]. On the other hand, anti-inflammatory effect has been observed in most herbs [17]. Bioactive components of herbs can reduce the biosynthesis of prostaglandins (mediators of inflammation) through their cyclooxygenase inhibitory activity. Decreased responses of inflammatory result in decreased migration of T cells to the villus epithelium [91]. The plantain and St. John's wort have strong cyclooxygenase inhibitory activity [92, 93]. This could be the main reason for the reduction of IEL in jejunum of piglets. In turn, the significant increase of IEL in the colon may be explained by herbs' polysaccharides, which can be as prebiotics for microbiota [49]. Intestinal microbiota can metabolize herbal molecules and produce a series of metabolites such as SCFAs, polyamines, organic acids, indole derivatives, and vitamins. Presumably, the increased IEL may be explained by an increased production of SCFA from fibre and polysaccharides in the colon. There are several positive effects of SCFAs in T cell population: they directly enhance the generation of T cells (Th1 and Th17), therefore increasing the ability to fight pathogens; they can encourage T cells to produce IL-10, which is an important aspect to prevent inflammatory responses and, under certain circumstances, can increase/decrease FoxP3+T cells as well [94]. Immune cells communicate with each other not only by directing cell-cell interactions but also by secreting factors [95]. The ability of *δγ* T cell to produce cytokines (IL-1*β* and TGF*β*) induces the spread of anti-inflammatory and antimicrobial CD4+ T cell in LP [79]. Therefore, the increased number of IELs can impact the distribution of T cells in LP.

## 5. Conclusion

Mild chronic diffuse enterocolitis may be a logical step caused by the immune system response to a new diet and circumstances. Therefore, the development of intestinal mucosal immunity is the most effective way to improve the gut health of piglets. Probiotic supplements improved the gut microbiota by increasing the count of *Lactobacillus* in the small and large intestines, while herbs had a tendency to decrease the count of *Enterobacteriaceae*. The improved microbiota of probiotics and the subsequent immune-stimulatory effect increased the number of IELs in the small intestine. It promoted the proliferation of enterocytes and goblet cells and increased the production of acidic mucins. In addition, the increased density of T cells in PPs and LP may indicate intense immune-modulatory effect to provide the immune system homeostasis and, at the same time, provide protection against pathogens. The inclusion of herbs in the piglet diet was characterized by a dual effect. Antimicrobial and anti-inflammatory properties of herbs are supposed to reduce immune-modulatory effect on the mucus of the small intestine, as evidenced by the shortage goblet cells and reduced number of T cells in LP and IELs. However, at the same time, herbs improved histomorphological parameters (the villus height, VH : CD ratio) and improved the proliferation of enterocytes and colonocytes. On the other hand, the increased distribution of T cells in PPs and in the large intestine suggests that some components of herbs can modulate the immune system. The effect of combination of probiotics and buckwheat bran was not found to be prospective, although some positive effects were observed in the large intestine. Additional studies are necessary in order to examine the inhibitory effect of buckwheat bran and probiotics combination. Further studies are needed for an improved understanding of probiotics and plant effects on the intestinal ecosystem by alteration of the microbiota, improved morphology of intestine, and stimulation of the immunology. The improved understanding will lead to an increased use of these alternatives in swine production.

## Figures and Tables

**Table 1:** Effects of probiotics and herbal products on intestinal microbiota (mean ± SEM).

Site and microbiota (log10 cfu·g^−1^)	Group				*p* value
	C	Pro	Pro+B	H	
*Jejunum*					
*Enterobacteriaceae*	5.29 ± 2.690	3.92 ± 0.563	3.04 ± 0.850	2.99 ± 0.671	0.4703
*Escherichia coli*	4.74 ± 2.210	3.54 ± 0.457	3.96 ± 0.826	2.99 ± 0.670	0.5938
*Lactobacillus* spp.	6.27 ± 0.815^b^	7.75 ± 0.242^a^	6.62 ± 0.140^ab^	5.98 ± 0.511^b^	0.0354
*Colon*					
*Enterobacteriaceae*	5.80 ± 0.804^x^	5.57 ± 0.290^xy^	4.00 ± 1.043^xy^	3.55 ± 0.279^y^	0.0937
*Escherichia coli*	4.86 ± 0.613	5.21 ± 0.256	3.88 ± 0.989	3.51 ± 0.293	0.2136
*Lactobacillus* spp.	6.48 ± 0.265^y^	7.99 ± 0.031^x^	7.12 ± 0.417^xy^	7.12 ± 0.375^xy^	0.0842

SEM: standard error of measurements. ^a,b^Mean values within a row with different superscript letters are significantly different (*p* < 0.05). ^x,y^Mean values within a row with different superscript indicate a tendency for difference between groups at 0.05 < *p* < 0.10.

**Table 2:** Effects of probiotics and herbal products on intestinal morphology in piglets (mean ± SEM).

Item	Group				*p* value
	C	Pro	Pro+B	H	
*Jejunum*					
Villus height (*μ*m)	357.37 ± 23.433^c^	416.87 ± 9.805^b^	400.79 ± 8.870^b^	482.87 ± 14.956^a^	<0.0001
Villus width (*μ*m)	146.75 ± 6.597^b^	176.83 ± 6.555^a^	168.64 ± 5.504^a^	168.32 ± 5.672^a^	0.0310
Crypt depth (*μ*m)	327.16 ± 21.484	306.03 ± 6.947	299.02 ± 7.942	290.51 ± 9.614	0.1625
VH : CD ratio	1.12 ± 0.051^c^	1.39 ± 0.042^b^	1.38 ± 0.049^b^	1.72 ± 0.068^a^	<0.0001
*Colon*					
Crypt depth (*μ*m)	461.31 ± 7.007^c^	533.98 ± 10.857^a^	525.90 ± 12.819^ab^	498.58 ± 5.692^b^	0.0002

SEM: standard error of measurements, VH: villus height, CD: crypt depth. ^a,b^Mean values within a row of different superscript letters are significantly different (*p* < 0.05).

**Table 3:** Mean ± SEM of the total number and density of goblet cells in the intestine of piglets.

Goblet cells	Group				*p* value
	C	Pro	Pro+B	H	
*Jejunum*					
n/villus	9.05 ± 0.809	10.88 ± 0.714	9.98 ± 0.481	9.55 ± 0.491	0.2330
GC/unit height (*μ*m)^*∗*^	0.03 ± 0.004^a^	0.03 ± 0.002^a^	0.03 ± 0.001^a^	0.02 ± 0.001^b^	0.0042
n/crypt	21.55 ± 1.550	23.43 ± 1.138	22.08 ± 0.966	20.75 ± 0.699	0.2761
GC/unit depth (*μ*m)^*∗*^	0.07 ± 0.007	0.08 ± 0.004	0.08 ± 0.004	0.08 ± 0.003	0.8101
*Colon*					
n/crypt	34.90 ± 1.610^b^	38.38 ± 1.118^a^	35.30 ± 1.222^ab^	34.35 ± 0.660^b^	0.0394
GC/unit depth (*μ*m)^*∗*^	0.08 ± 0.004	0.07 ± 0.002	0.07 ± 0.002	0.07 ± 0.003	0.2751

GC: goblet cells, SEM: standard error of measurements. ^a,b^Mean values within a row of different superscript letters are significantly different (*p* < 0.05). ^*∗*^Number of goblet cells per unit of villus height or crypt depth.

**Table 4:** Mean ± SEM of the total number and density of PAS^+^ and AB^+^ goblet cells in the intestine of piglets.

Parameters	C	Pro	Pro+B	H	*p* value
*Villus (jejunum)*					
AS^+^ n/villus	8.30 ± 0.645	9.63 ± 0.685	9.48 ± 0.510	10.00 ± 0.621	0.4221
PAS^+^ GC/unit height (*μ*m)^*∗*^	0.03 ± 0.003	0.02 ± 0.001	0.02 ± 0.001	0.02 ± 0.001	0.2158
AB^+^ n/villus	8.05 ± 0.720^b^	10.78 ± 0.686^a^	9.45 ± 0.556^ab^	8.37 ± 0.512^b^	0.0116
AB^+^ GC/unit height (*μ*m)^*∗*^	0.02 ± 0.003^a^	0.03 ± 0.002^a^	0.02 ± 0.001^a^	0.02 ± 0.002^b^	0.0004
crypt (jejunum)					
PAS^+^ n/crypt	20.30 ± 1.244	21.98 ± 0.708	21.83 ± 0.697	21.53 ± 0.617	0.5588
PAS^+^ GC/unit depth (*μ*m)^*∗*^	0.07 ± 0.006	0.07 ± 0.003	0.08 ± 0.004	0.08 ± 0.003	0.3131
AB^+^ n/crypt	21.10 ± 0.932^ab^	22.53 ± 1.005^a^	20.38 ± 0.690^ab^	19.13 ± 0.687^b^	0.0240
AB^+^ GC/unit depth (*μ*m)^*∗*^	0.07 ± 0.005	0.08 ± 0.004	0.07 ± 0.003	0.68 ± 0.003	0.2604
*Crypt (colon)*					
PAS^+^ n/crypt	29.35 ± 1.091^b^	35.60 ± 1.182^a^	36.15 ± 1.057^a^	34.30 ± 1.450^a^	0.0082
PAS^+^ GC/unit depth (*μ*m)^*∗*^	0.06 ± 0.003	0.07 ± 0.002	0.07 ± 0.002	0.07 ± 0.005	0.4529
AB^+^ n/crypt	31.20 ± 1.423^y^	35.78 ± 0.655^x^	34.80 ± 1.181^xy^	33.48 ± 1.312^xy^	0.0841
AB^+^ GC/unit depth (*μ*m)^*∗*^	0.07 ± 0.004	0.07 ± 0.002	0.07 ± 0.003	0.07 ± 0.003	0.9939

PAS^+^: Periodic Acid-Schiff positive cells, AB^+^: Alcian blue positive cells, GC: goblet cells, SEM: standard error of measurements. ^a,b^Mean values within a row of different superscript letters are significantly different (*p* < 0.05). ^x,y^Mean values within a row with different superscript indicate a tendency for difference between groups at 0.05 < *p* < 0.10. ^*∗*^Number of goblet cells per unit of villus height or crypt depth.

## Data Availability

The data used to support the findings of this study are available from the corresponding author upon request.
